# A high-throughput cell-based assay to screen for drugs affecting PfEMP1 transport to RBC surface

**Published:** 2011-01-10

**Authors:** Min-Je Ku, Fernando M Dossin, Michael AE Hansen, Minjung Ma, Auguste Genovesio, Lucio Freitas-Junior

**Affiliations:** 1 Center for Neglected Diseases Drug Discovery (CND3)-Malaria Drug Discovery, Institut Pasteur Korea, Seongnam-si, Korea; 2 Center for Core Technologies-Image Mining, Institut Pasteur Korea, Seongnam-si, Korea

## 

Upon infection of human red blood cells (RBCs), the malaria parasite *Plasmodium falciparum* starts to modify its host cell by exporting parasite-encoded proteins to the RBC cytosol and membranes. One of these proteins, the *P*. *falciparum* erythrocyte membrane protein 1(PfEMP1) is a major virulence factor. It is expressed on the infected RBC surface and mediates cytoadherence to several host endothelium receptors, enabling parasite escape from spleen clearance but giving rise to severe hemorrhagic complications of malaria.

Here we show the development of an assay to screen for drugs affecting either PfEMP1 synthesis or transport to RBC surface. In this assay, we incubate synchronized young parasites with compounds in 384-well plate. After 20 h incubation, parasites are transferred to another plate containing human placenta BeWo cells and are allowed to bind to BeWo cells for 1 h. Unbound RBCs are washed out and BeWo cells and remaining bound RBC are fixed and stained respectively by the use of a fluorescent anti-Glycophorin A antibody and the DNA dye Syto60. Images are acquired in an automated, high-throughput confocal microscope platform and analyzed by an algorithm specialized for this assay. We expect that our screening to result in the discovery of anti-malarial drugs inhibiting PfEMP1 transport or assembly at the RBC surface and thus disabling a major parasite escape mechanism.

